# Comparison of Outcomes and Survival of Two Cohorts of Patients with Simultaneous Pancreas-Kidney Transplantation: A Retrospective Cohort Study in a Latin American Hospital

**DOI:** 10.1155/2023/2734072

**Published:** 2023-06-15

**Authors:** Iván Darío Martin-González, Luis Manuel Barrera-Lozano, Oscar Alonso Villada-Ochoa, Jaime Alberto Ramírez-Arbeláez, Néstor Alfonso López-Pompey, Dabely América Palacios, Jorge Andrés Becerra-Romero, Cristian Leonardo Muñoz, Daniel González-Arroyave, Carlos M. Ardila

**Affiliations:** ^1^Fundación Clínica Abood Shaio, Universidad de Antioquia, Medellín, Colombia; ^2^Digestive Diseases and Transplant Functional Unit, Hospital San Vicente Fundación, Rionegro, Colombia; ^3^Faculty of Medicine, Universidad de Antioquia, Medellín, Colombia; ^4^Research Unit, Hospital San Vicente Fundación, Rionegro, Colombia; ^5^Universidad de Antioquia, Medellín, Colombia

## Abstract

**Background:**

Simultaneous pancreas-kidney transplantation (SPKT) is a complex and demanding procedure with a considerable risk of morbidity and mortality. Advances in surgical techniques and organ preservation have introduced changes in care protocols. Two cohorts of patients receiving SPKT with two different protocols were compared to determine overall survival and pancreatic and renal graft failure-free survival.

**Methods:**

This retrospective observational study was conducted in two cohorts of SPKT recipient patients that underwent surgery between 2001 and 2021. Outcomes were compared in transplant patients between 2001 and 2011 (cohort 1; initial protocol) and 2012-2021 (cohort 2; improved protocol). In addition to the temporality, the cohorts were defined by a protocolization of technical aspects and medical management in cohort 2 (improved protocol), compared to a wide variability in the procedures carried out in cohort 1 (initial protocol). Overall survival and pancreatic and renal graft failure-free survival were the primary outcomes. These outcomes were determined using Kaplan-Meier survival analysis and the log-rank test.

**Results:**

Fifty-five SPKT were performed during the study period: 32 in cohort 1 and 23 in cohort 2. In the survival analysis, an average of 2546 days (95% CI: 1902-3190) was found in cohort 1, while in cohort 2, it was 2540 days (95% CI: 2100-3204) (*p* > 0.05). Pancreatic graft failure-free survival had an average of 1705 days (95% CI: 1037-2373) in cohort 1, lower than the average in cohort 2 (2337 days; 95% CI: 1887-2788) (*p* = 0.016). Similarly, renal graft failure-free survival had an average of 2167 days (95% CI: 1485-2849) in cohort 1, lower than the average in cohort 2 (2583 days; 95% CI: 2159-3006) (*p* = 0.017).

**Conclusions:**

This analysis indicates that pancreatic and renal graft failure-free survival associated with SPKT decreased significantly in cohort 2, with results related to improvements in the treatment protocol implemented in that cohort.

## 1. Introduction

In the different types of diabetes mellitus (DM), some mechanisms lead to chronic hyperglycemia, ranging from an absolute deficit in insulin secretion (as in type 1 DM) to resistance to insulin (as occurs in DM type 2), among other mechanisms. DM can affect people of all ages, ethnic groups, sex, and socioeconomic levels. Currently, around 500 million people in the world live with the disease, which contributes to a high burden of morbidity and mortality [1].

The prevalence of DM has increased markedly in Latin America. The underdiagnosis of this condition is notable in this region, in addition to the risk factors [2]. Specifically in Colombia, an increase in the prevalence of type 2 DM has been reported, ranging between 4% and 8% depending on the demographic zone and the reference population group. A higher incidence is observed in women, in urban areas, and in the population with medium-low incomes. On the other hand, the incidence of type 1 DM is low (3-4 per 100,000 children under 15 years of age), and a prevalence of 0.07% is estimated [3].

The relationship between DM and chronic kidney disease (CKD) is established, with diabetic nephropathy being the main cause of CKD. CKD requiring renal replacement therapy represents about 45% of cases; other associated factors such as obesity, arterial hypertension, and ischemic heart disease are added [4].

Renal transplantation is widely recognized as the best therapeutic alternative for most CKD patients. Likewise, simultaneous pancreas-kidney transplantation (SPKT) is considered to represent the best treatment option for patients with CKD, with an indication for kidney transplantation and type 1 DM [5]. In this particular population, several studies have evaluated different outcomes comparing four groups of patients: patients who remain on the waiting list, patients who receive a deceased-donor kidney transplant, patients who receive a living-donor kidney transplant, and those who receive deceased-donor SPKT [6, 7].

SPKT is a complex and demanding procedure with a considerable risk of morbidity and mortality; however, in terms of long-term patient survival (greater than 10 years), the population with the best results corresponds to SPKT recipients. Moreover, the initial increase in perioperative mortality is offset during follow-up by the benefits of better glycemic control (in the long term) with a lower probability of dying from DM complications [6]. In patients with type 2 DM and chronic kidney disease, the role of the SPKT continues to be controversial; however, recent evidence highlights important benefits in patients selected from this population, showing results that are not inferior to those exhibited by recipients with type 1 DM [7].

Over the years, advances in surgical techniques, immunosuppression, and preservation technology have emerged, improving SPKT outcomes; however, several concerns in this regard, such as the selection of the donor and recipient, the choice of the optimal procedure, organ procurement, preservation techniques, and ideal immunosuppression, have remained latent [8]. In fact, different protocols have been described in the literature [9–13]. Despite the dissemination of different clinical guidelines focused on specific aspects of pancreas transplants [9–11], it was not until the end of 2019 that the first international consensus was reached to holistically evaluate pancreas transplantation to establish evidence-based guidelines [8, 12]. For these reasons, it is important to compare care protocols to assess the possible improvements they have caused.

In the present study, two cohorts of patients receiving SPKT with two different protocols were compared to determine overall survival and pancreatic and renal graft failure-free survival. In addition to the temporality, the cohorts were defined using a protocolization of technical aspects and medical management in cohort 2 (improved protocol), compared to a wide variability in the procedures carried out in cohort 1 (initial protocol).

## 2. Materials and Methods

### 2.1. Study Design

This single-center, retrospective observational study analyzes the information of a group of patients who received SPKT and underwent surgery between 2001 and 2021 at the Hospital San Vicente Fundación, Rionegro, Colombia. Records of adult patients with CKD who presented type 1 DM and were SPKT recipients were included. The sample included all the records of patients who met the eligibility criteria. The study protocol was approved by the Research Ethics Committee of the Hospital San Vicente Fundación, Rionegro, Colombia (written consent was obtained by the review board). Moreover, the bioethical recommendations of the Declaration of Helsinki were followed. This study also complies with the principles established by the Declaration of Istanbul on Organ Trafficking and Transplant Tourism.

#### 2.1.1. Study Variables

Overall survival and pancreatic and renal graft failure-free survival were the primary outcomes. Moreover, the following features were considered as secondary outcomes: cold pancreatic ischemia, cold kidney ischemia, duration of surgery, early outcomes at 30 days (transfusion at surgery, time in intensive care unit (ICU), reintervention, delayed graft function, postoperative transfusion, infection, pancreatic and kidney graft function, pancreatic graft thrombosis, and death), and late outcomes (follow-up time, death, and pancreatic and kidney graft failure). The age of the donor and the characteristics of the recipient were also evaluated (age, gender, weight, height, time of the recipient's diagnosis of diabetes, and time on dialysis of the recipient).

Pancreatic graft failure was defined as requiring exogenous insulin therapy, explantation, or death of the recipient. Renal graft failure was defined as a return to permanent dialysis therapy, explantation, or death of the recipient. Early graft failure was defined as that which occurs in the first month after transplantation.

In addition to the temporality, the cohorts were defined by a protocolization of technical aspects and medical management in cohort 2, compared to a wide variability in the procedures carried out in cohort 1. In cohort 1 (initial protocol), the extraction of the pancreas was performed in a block with the liver and was later separated during bench surgery. Moreover, vascular reconstruction in bench surgery was performed with portal vein grafting and long arterial grafts. Unlike cohort 1, in cohort 2 (improved protocol), aspects such as *in situ* extraction and hot dissection, arterial vascular reconstruction with the Y graft to the superior mesenteric artery, the splenic artery of the graft (used in only 1/3 of the cases in cohort 1), systemic venous drainage (using a portocaval anastomosis), a small duodenal segment with enteric exocrine drainage (using a laterolateral duodenojejunostomy), use of an immunosuppression scheme that included thymoglobulin induction (in all patients together with steroids, anticalcineurin, and mycophenolate), and close monitoring of glycemia in the postoperative period were implemented. Hyperglycemia and insulin requirement were considered as indicators of graft vascular complications.

The information was obtained from secondary sources (database of the transplant program of the Hospital San Vicente Fundación, Rionegro, presurgical clinical history, surgical notes, and evolutions) that remain in the institutional clinical history application. The information was consolidated in a format predesigned for the study. The data extraction and analysis procedures were blinded in such a way that the information related to the data of the individuals and the respective cohort was known after the analysis was completed. Once the survey of all patients was obtained, the population was divided into two cohorts, one that received the transplant protocol used between 2001 and 2011 (initial protocol) and the other cohort that received the protocol implemented between 2012 and 2021 (improved protocol).

### 2.2. Statistical Analysis

For the description of the demographic and clinical characteristics of the patients, absolute and relative frequencies were reported. For the quantitative variables, the assumption of normality was verified with the Shapiro-Wilk test. Variables with a normal distribution were reported using the arithmetic mean with the respective standard deviation. Otherwise, the median and interquartile range (IQR; 25th-75th percentile) were reported.

An exploratory analysis was carried out to establish the differences in the variables of interest, between the patients receiving combined transplants operated on before the year 2011 (cohort 1; initial protocol) and after the year 2012 (cohort 2; improved protocol). The above was carried out using the Mann–Whitney test for quantitative variables and Pearson's chi-square test for qualitative variables. Statistical significance was defined as *p* < 0.05. Overall survival, renal graft failure-free survival, and pancreatic graft failure-free survival were determined using Kaplan-Meier survival analysis. Survival between the two study cohorts was compared using the log-rank test. Confidence intervals (CI) of 95% were reported. The assumptions of these analyses were considered. The analysis was performed with a statistical package (SPSS v 24 IBM).

## 3. Results

Between 2001 and 2021, 55 SPKT were performed in 55 patients with a median age of 35 years (IQR: 30-40 years). A total of 58.2% of the patients were male, with a mean of 20 years in the time elapsed between the diagnosis of diabetes and renal transplantation, and 2 years from the start of renal replacement therapy and transplantation (Table 1).

Thirty-two patients received the transplant between 2001 and 2011 (cohort 1; initial protocol) and 23 between 2012 and 2021 (cohort 2; improved protocol). The median age was higher in cohort 2 compared to cohort 1 (37 versus 31 years, *p* = 0.013), while the proportion of men transplanted in cohort 2 tended to be higher, but without statistically significant differences (69.6% versus 50%; *p* = 0.147).

Kidney graft cold ischemia time was 9 hours versus 10 hours, in cohort 1 and cohort 2, respectively. Anthropometric characteristics, disease times, donor age, cold ischemia of the pancreas, and surgical time did not show significant differences (Table 2).

The analysis of the results at 30 days revealed a trend towards a lower requirement for transfusion therapy in the intraoperative period in the second cohort (68.8% versus 52.2%), while the use of blood products, in the postoperative period, showed significant differences between cohorts 1 and 2 (81.3% and 47.8%, respectively). A total of 62.5% of the patients in cohort 1 required at least one reoperation compared to 26.1% in cohort 2 (*p* = 0.008).

Delayed kidney graft function occurred in 25% of the cases in cohort 1 and 4.3% in cohort 2 (*p* = 0.04). Infection was documented in 53.1% and 8.7% in cohorts 1 and 2, respectively, with statistically significant differences (*p* = 0.001) (Table 3).

The follow-up period of cohorts 1 and 2 was 716 days (IQR 298-1513) and 910 days (IQR 450-1666), respectively. Death-censored graft failure was significantly higher in cohort 1 (10/32) versus cohort 2 (2/23) (*p* = 0.001). Renal graft failure occurred in 15% of cohort 1 and 4.3% of cohort 2, while pancreatic graft failure occurred in 31.3% of patients in cohort 1 and 13% in cohort 2 (Table 4).

**Figure 1:**
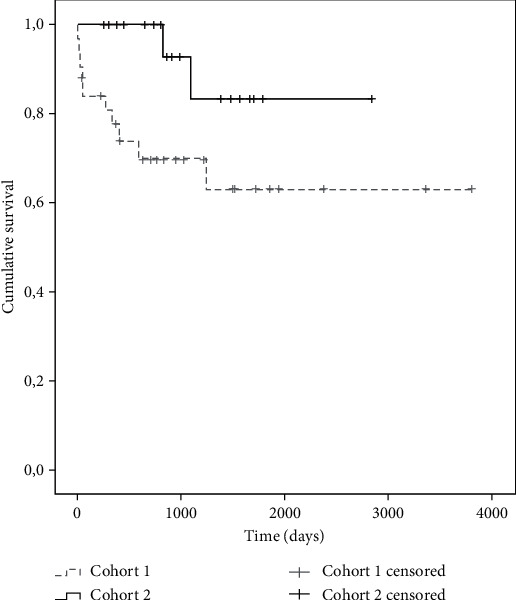
Survival in years according to the cohort of patients with simultaneous pancreas-kidney transplantation.

**Figure 2:**
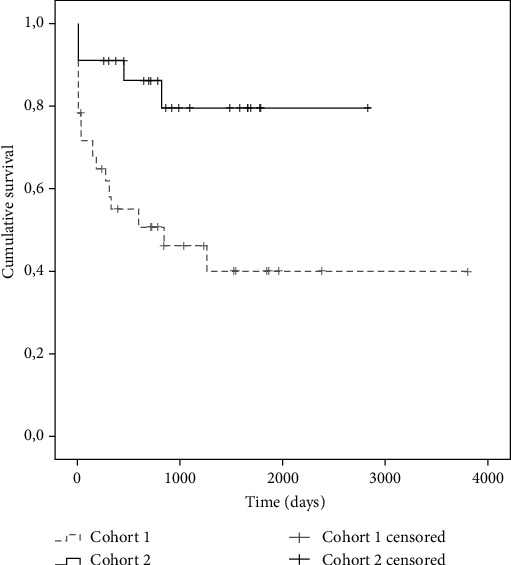
Failure-free survival of transplanted pancreatic graft according to the patient cohort.

**Figure 3:**
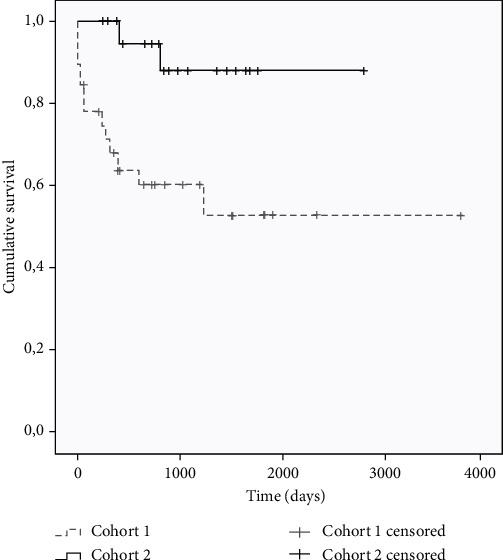
Renal graft failure-free survival according to the patient cohort.

Considering the primary results, the following was observed. In the survival analysis, an average of 2546 days (95% CI: 1.902-3.190) was found in cohort 1, while it was 2540 days in cohort 2 (95% CI: 2100-3204), without statistically significant differences (*p* = 0.112) (Figure 1). Pancreatic graft failure-free survival was evaluated between both cohorts. An average of 1705 days (95% CI: 1037-2373) was found in cohort 1, lower than the average in cohort 2, which was 2337 days (95% CI: 1887-2788), with statistically significant differences (*p* = 0.016). The Kaplan-Meier curves are observed in Figure 2. Renal graft failure-free survival between both cohorts was also evaluated (Figure 3). An average of 2167 days (95% CI: 1485-2849) was found in cohort 1, lower than the average in cohort 2, which was 2583 days (95% CI: 2159-3006), with statistically significant differences (*p* = 0.017).

## 4. Discussion

In this study, two cohorts of patients that received SPKT were evaluated. When comparing the two cohorts, a lower percentage of reoperations, infections, and graft failure and a lower proportion of mortality were observed in cohort 2 (improved protocol).

A recent international consensus concluded that pancreatic transplantation could improve patient survival in the long term, providing a dramatic improvement in the quality of life of recipients; furthermore, pancreatic transplantation can improve the course of chronic complications of diabetes, depending on the severity [8]. Thus, the advantages of the intervention seem to compensate for the potential disadvantages.

The extension of the indication to selected patients with type 2 DM and the recent evidence (on improvement over time in outcomes and long-term benefits) have positioned SPKT at the level of the best treatment alternatives previously available, such as living-donor kidney transplantation and optimal medical management of diabetes [14, 15].

Patients with diabetes and chronic kidney disease experience excessive morbidity and mortality; however, SPKT has shown improved outcomes, such as graft survival at 1, 3, and 5 years. These results are comparable to that of cardiac, hepatic, and renal grafts [16]. The results presented here show this trend.

According to information from the International Pancreas Transplant Registry (IPTR), the survival of patients receiving pancreas transplants in the West is greater than 95% at 1 year and 90% at 3 years, while the 1-year graft survival for SPKT recipients is 85%. This data is close to that found in the second cohort of our center and seems to be maintained over time, at least until the scope of follow-up in this study. It has been established that the half-life of a pancreatic graft in the context of SPKT is over 14 years. The variable with the greatest impact on this improvement seems to be pancreatic graft survival at one year, which in turn has been favored mainly by a decrease in early graft loss, usually related to perioperative complications [17]. Similarly, our data regarding the survival of patients and grafts show a considerable impact in the first thirty days, with subsequent stabilization towards the first year. We consider that the differences between the curves of the cohorts reported are probably related to the decrease in mortality and morbidity in the early postoperative period; this is due to the improvement of some aspects of the donation, extraction, implant, and perioperative management process, which are important to note.

Donor selection is considered a critical shortcoming of pancreatic donors. In this regard, it is recommended that the possibility of extracting the pancreatic graft be analyzed in each potential deceased donor. In our context, the low number of recipients on the waiting list (partly because some potential recipients with a clear indication are listed only for kidney transplantation) allows transplant centers to be rigorous in the selection of pancreas donors. As shown in the two cohorts analyzed, the donors of the grafts for combined transplantation did not exceed 30 years of age on average.

In the literature, there are tools available such as the “preprocurement pancreas suitability score (P-PASS)” [18] and the “Pancreas Donor Risk Index (PDRI)” [19], which have established certain donor factors that impact outcomes. These include age, gender, ethnicity, body mass index, cause of death, renal function, natremia, length of stay in the ICU, and cardiac arrest, among others. The performance of these tools has been evaluated in different studies. The P-PASS was able to assess and predict the risk of graft loss in the first month, while the highest score, using the PDRI scale, has been correlated in several studies with the risk of graft loss at one year [20]. In this study, the variables age and gender were documented among the factors included in the scales. Based on our experience, we also consider it important to seek control of factors that may prolong cold ischemia times beyond 12 hours.

Regarding the extraction and preservation phase, we have found convenient *in situ* and hot dissection of the hepatoduodenal ligament. This dissection is demanding and time-consuming; however, we consider that this procedure is safe with the potential for less bleeding during reperfusion, with a reduction in the need for reinterventions for this cause, as documented in the comparison between cohorts.

For preservation, the Belzer-UW® solution was used, instilled through the aorta and portal vein; other solutions have been used with dissimilar results, some of them markedly unfavorable for other solutions [15, 21]. Among the cohorts analyzed, despite the wide temporal difference between them, there were no significant differences in ischemia times, accounting for the persistence of considerable logistical challenges.

In bench surgery, a standard technique of reconstruction with the arterial Y graft and dissection for portal elongation without the vein graft was applied to all recipients in the second cohort. We believe that this approximation could be related to the differences observed in the risk of pancreatic graft thrombosis.

A right retroperitoneal position of the graft was used, with systemic venous drainage. The literature contains variable reports regarding the benefits and disadvantages of drainage to the portal circulation versus drainage to the systemic circulation; however, no technique has been established with significant clarity [8, 15, 21]. Arterial reconstruction is achieved by anastomosing the proximal end of the graft in Y, to the right common iliac artery. Exocrine drainage is achieved via manual laterolateral duodenum-jejunal anastomosis; upper enteric drainage was considered, in line with the literature [8, 22, 23]. Some authors have suggested the benefits of exocrine drainage through duodenum-duodenum anastomosis that allows access to the graft through endoscopy and endosonography, a matter that would facilitate follow-up of the graft while obtaining a more fixed position. This is an aspect that could reduce the incidence of graft vascular complications [22–24].

SPKT is a technically demanding procedure; it has been considered the solid organ transplant with the highest rate of complications, especially in the first month after transplantation [5, 15, 17]. The analysis of this study shows results that have improved significantly over time, including reduced transfusion requirements, shorter ICU stay, lower incidence of delayed renal graft function, lower risk of infections, better early renal and pancreatic graft survival, and lower mortality. This alternative is positioned as a therapeutic option to be offered in our setting, in the context of promising long-term follow-up results, and especially the expansion of the indications reported in the literature [8, 14, 15].

The restriction of this treatment to the subgroup of patients with type 1 DM and advanced kidney disease has been challenged by different groups, with long-term results exceeding optimistic expectations for a selected group of patients with type 2 DM [25, 26]. According to data from the Scientific Registry of Transplant Recipients (SRTR), the number of patients who entered the waiting list for an SPKT was higher in 2019 and 2020 than the figure for any previous year, since 2012 (despite the impact of the pandemic). On the other hand, the proportion of patients with type 2 DM exceeded 20%, an amount that has doubled over the last five years [27]. Some centers in Latin America have used this intervention beyond the widely accepted initial indication, finding promising initial results [28].

In general, with some exceptions [29, 30], the practice of pancreas transplantation in the region is located in a few care centers with limited volume; however, it is a development opportunity with potential benefits for a considerable number of patients.

Pancreatic transplantation is the only method of replacing complete pancreatic function in insulin-dependent patients [31, 32]. The advances in this intervention mean that it not only rivals but often exceeds the results of other solid organ transplants [32, 33]. Particularly favorable results have been observed among diabetic patients with progressive deterioration of renal function, in predialysis [34]. Some alternatives such as pancreas transplantation after a kidney (especially when the renal graft comes from a living donor) have shown considerable benefits in terms of survival of the patient and the grafts, suggesting a protective effect of the pancreatic graft on the renal graft compared with the conventional management of a metabolic disease [34, 35]. The length of time on dialysis of the patients, especially of the most recent cases of cohort 2, has tended to be lower due to early patient referral. Advances in the safety profile of this intervention in our environment have become known in the local medical community, which has favored its indication in more patients.

This study represents the largest series of patients with SPKT in Colombia, with analysis of early outcomes, renal and pancreatic graft failure-free times, and recipient survival. In the future, prospective analytical and multicenter studies should be carried out to evaluate other factors not considered in this work, including specific characteristics of donors and recipients, immunosuppression schemes, other outcomes, and especially the quality of life.

The main limitation of this study is related to the retrospective nature of its design, where a causal relationship is not established; however, cohort studies are among the highest levels of scientific evidence [36–38].

In conclusion, simultaneous pancreas-kidney transplantation is a demanding procedure with great benefits in the medium and long term for patients with advanced chronic kidney disease and diabetes mellitus. However, better results were observed in cohort 2 (improved protocol) in terms of reoperation, infection, delayed graft function, and death-censored graft failure. Moreover, this study denoted that pancreatic and renal graft failure-free survival associated with SPKT decreased significantly in cohort 2, with results related to improvements in the treatment protocol implemented in that cohort.

## Figures and Tables

**Table 1:** Characteristics of combined pancreas-kidney transplant recipients 2001-2021.

Characteristic	*N*	%
Age (years), median (interquartile range)	35 (30-40)	
Gender		
Male	32	58.2
Female	23	41.8
Weight (kilograms), median (interquartile range)	58 (51-65)	
Height (centimeters), median (interquartile range)	163 (154-168)	
Time diagnosed with diabetes mellitus (years), median (interquartile range)	20 (17-27)	
Recipient dialysis time (years), median (interquartile range)	2 (1-4)	

**Table 2:** Characterization of the patient cohorts with combined pancreas-kidney transplantation 2001-2021.

Variable	Cohort 1	Cohort 2	*p* value
	*n* = 32	*n* = 23	
Age (years), median (interquartile range)	31 (27-38)	37 (32-44)	0.01
Gender, *n* (%)			
Male	16 (50)	16 (69.6)	0.14
Female	16 (50)	7 (30.4)	
Height (centimeters), median (interquartile range)	163 (154-167)	162 (157-173)	0.18
Weight (kilograms), median (interquartile range)	57 (50.2-63.5)	60 (53-71)	0.22
Time with diabetes mellitus (years), median (interquartile range)	20 (16-23.5)	22 (20-29)	0.03
Recipient dialysis time (years), median (interquartile range)	2 (1-4)	2 (1-4)	0.82
Donor age (years), median (interquartile range)	21.5 (19-27.7)	21.5 (18-33.7)	0.88
Pancreatic cold ischemia (hours), median (interquartile range)	8 (7-10)	8.6 (7.7-9.4)	0.61
Kidney cold ischemia (hours), median (interquartile range)	9 (7-11.2)	10 (8.8-11.6)	0.05
Surgery duration (hours), median (interquartile range)	4.7 (4-6.5)	5 (4.5-5)	0.93

**Table 3:** Early outcomes (at 30 days) of the cohorts of patients with combined pancreas-kidney transplantation.

Variables	Cohort 1	Cohort 2	*p* value
	*n* = 32	*n* = 23	
Transfusion, *n* (%)			
Yes	22 (68.8)	12 (52.2)	0.21
No	10 (31.3)	11 (47.8)	
Time in ICU (days), median (interquartile range)	3.5 (2-7.7)	5 (3-8)	0.27
Reoperation, *n* (%)			
Yes	20 (62.5)	6 (26.1)	0.008
No	12 (37.5)	17 (73.9)	
Delayed graft function, *n* (%)			
Yes	8 (25)	1 (4.3)	0.04
No	24 (75)	22 (95.7)	
Postoperative transfusion, *n* (%)			
Yes	26 (81.3)	11 (47.8)	0.009
No	6 (18.8)	12 (52.2)	
Infection, *n* (%)			
Yes	17 (53.1)	2 (8.7)	0.001
No	15 (46.9)	21 (91.3)	
Pancreatic graft thrombosis			
Yes	6 (18.7)	2 (8.7)	0.29
No	26 (81.3)	21 (91.3)	
Kidney graft failure, *n* (%)			
Yes	5 (15.6)	0	0.04
No	27 (84.4)	23 (100)	
Pancreatic graft failure, *n* (%)			
Yes	7 (21.9)	2 (8.7)	0.27
No	25 (78.1)	21 (91.3)	
Death, *n* (%)			
Yes	2 (6.3)	0	0.22
No	30 (93.8)	23 (100)	

**Table 4:** Late outcomes in cohorts of patients with combined pancreas-kidney transplantation.

Variables	Cohort 1	Cohort 2	*p* value
	*n* = 32	*n* = 23	
Follow-up time (days), median (interquartile range)	716 (298-1513)	910 (450-1666)	0.18
Death, *n* (%)			
Yes	10 (31.3)	2 (8.7)	0.04
No	22 (68.8)	21 (91.3)	
Kidney graft failure, *n* (%)			
Yes	13 (40.6)	1 (4.3)	0.03
No	19 (59.4)	22 (95.7)	
Pancreatic graft failure, *n* (%)			
Yes	10 (31.3)	5 (21.7)	0.01
No	22 (68.8)	18 (78.3)	

## Data Availability

The datasets used and/or analyzed during the present study are available from the corresponding author upon reasonable request.
